# Quantification of macular perfusion using optical coherence tomography angiography: repeatability and impact of an eye-tracking system

**DOI:** 10.1186/s12886-018-0789-z

**Published:** 2018-05-24

**Authors:** Maged Alnawaiseh, Cristin Brand, Eike Bormann, Cristina Sauerland, Nicole Eter

**Affiliations:** 1 Department of Ophthalmology, University of Muenster Medical Center, Albert-Schweitzer-Campus 1, Building D15, 48149 Muenster, Germany; 2 Centre of Reproductive Medicine and Andrology, University of Muenster, Muenster, Germany; 3 Institute of Biostatistics and Clinical Research, University of Muenster, Muenster, Germany

**Keywords:** Eye-tracking system, Macular perfusion, Optical coherence tomography angiography

## Abstract

**Background:**

The aim of the study was to evaluate the impact of integration of the eye-tracking system (ET) on the repeatability of flow density measurements using optical coherence tomography (OCT) angiography.

**Methods:**

20 healthy subjects were included in this study. OCT-angiography was performed using RTVue XR Avanti (Optovue Inc., Fremont, California, USA). The macula was imaged using a 3 × 3 mm scan twice with and twice without activation of the ET. Flow density data of the macular in the superficial and deep OCT angiograms were extracted and analyzed.

**Results:**

The difference between the flow density (whole en face) in the first session and second session with and without ET was statistically non-significant (with ET: superficial retinal OCT angiogram: *p* = 0.50; deep retinal OCT angiogram: *p* = 0.89; without ET: superficial retinal OCT angiogram: *p* = 0.81; deep retinal OCT angiogram: *p* = 0.24). There was no significant difference in the coefficients of repeatability for measurements with and without ET in the superficial retinal OCT angiogram (adjusted *p*-value = 0.176), whereas the difference was significant for the deep retinal OCT angiogram (adjusted *p*-value = 0.008).

**Conclusions:**

Integration of the ET improved the repeatability of flow density measurements in the deep OCT angiogram; this needs to be considered when evaluating the long-term changes of flow density and when comparing data of different studies and different devices.

## Background

Optical coherence tomography angiography (OCT angiography) was first reported by Makita et al. using Doppler OCT [1]. OCT angiography is a novel technology allowing layer-specific visualization of normal chorioretinal vasculature and neovascularizations without the need for intravenously injected fluorescent dyes [2–5]. The visualization of normal vessels and pathological neovascularization using OCT angiography has been evaluated in different retinal or choroidal neovascular diseases such as chronic central serous chorioretinopathy, age-related macular degeneration, retinal vein occlusion, diabetic retinopathy, and retinal arterial macroaneurysms and also in different animal models. This facility of OCT angiography imaging has been described as a useful tool in the diagnosis and follow-up of such diseases [3–8].

Another promising aspect of OCT angiography, which could be very useful in clinical practice and for clinical and experimental research, is the ability to quantify blood flow. Various recent studies have evaluated flow density (FD) in normal subjects and in different retinal pathologies as well as pathologies of the optic nerve head [9–16].

In the past, OCT angiography and measurement of blood flow have usually been evaluated using the RTVue XR Avanti with AngioVue (Optovue Inc., Fremont, California, USA), while split-spectrum amplitude-decorrelation angiography (SSADA) was used to extract the OCT angiography information. A new development of the RTVue XR Avanti is the integration of an eye-tracking system (ET) with OCT angiography imaging. The newly introduced software update of this device enables the ET to be activated or deactivated on imaging.

The aim of the study was to evaluate the impact of integration of the eye-tracking system on the repeatability of flow density measurements.

## Methods

This prospective study included 20 eyes of 20 healthy volunteers with no history of any ocular or systemic disease or ocular surgery. Before performing OCT angiography imaging, the study protocol was explained in detail and each participant signed an informed consent form. The study followed the tenets of the Declaration of Helsinki.

After performing slit-lamp biomicroscopy and funduscopy of the macula and the optic nerve head (ONH), all participants were asked to rest for five minutes. The macula was imaged using a 3 × 3 mm scan twice with and twice without activation of the eye-tracking system; the sequence (with ET and without ET) was randomly defined. *Flow density data of the macula in the superficial retinal OCT* angiograms (from the inner limiting membrane with an offset of 3 μm to the inner plexiform layer with an offset of 15 μm) and deep OCT angiograms (segmented with an inner boundary at 15 μm beneath the inner plexiform layer and the outer boundary at 70 μm beneath the inner plexiform layer) were extracted and analyzed.

### OCT angiography

The teleological principles of OCT-angiography have been described in detail in a number of previous studies [3–6, 13–15]. Briefly, OCT scans of a defined region of the retina or of the optic nerve head are performed several times, and the OCT images analyzed and examined for changes. Static tissue shows little or no change, whereas blood flow will result in changes between successive images [17].

In the present study OCT angiography imaging was performed using the RTVue XR Avanti with AngioVue (Optovue Inc., Fremont, California, USA). The system has an A-scan rate of 70,000 scans per second, using a light source centered on 840 nm and a bandwidth of 45 nm. Each OCT angiography volume contained 304 × 304 A-scans with two consecutive B-scans that were captured at each fixed position before proceeding to the next sampling location. The SSADA algorithm is used to identify blood flow and to generate the OCT angiograms [3–6].

Only one eye of each participant was randomly included in the study. OCT angiography imaging was performed under the same setting by the same examiner in the same location, and only images with a signal strength index of ≥60 were included. In cases with significant motion artifacts or poor signal strength, the resulting OCT angiography image contains lines or gaps. Images with these artifacts were not included in the study.

### Statistical analysis

Microsoft Excel 2010 was used for data management. Statistical analyses were performed using IBM SPSS® Statistics 23 for Windows (IBM Corporation, Somers, NY, USA). Data are presented as mean ± standard deviation, minimum and maximum. The mean of the two measurements before and after activation of the eye-tracking system were compared using t-tests for paired data. All *p*-values below 0.05 were considered significant.

In order to assess the repeatability between the first and second scan, the intraclass correlation coefficient (ICC(2,1)) as well as the coefficient of repeatability (CR) were calculated [18, 19]. A two-sided paired t-test was used to compare the coefficients of repeatability for measurements with and measurements without eye tracker for both the superficial and deep retinal OCT angiograms. For those two tests a Bonferroni correction was applied to adjust the *p*-values. Bland Altman plots were used to show the agreement between the two measurements for each subject. In these plots the difference of the two measurements is plotted against their mean. Since differences can be assumed to be normally distributed, one would expect 95% of the observed differences to lie within the limits of agreement (mean-1.96*SD, mean + 1.96*SD) [20].

## Results

20 eyes of 20 subjects were included in the study (age = 33 ± 2.5 (20–56) years). The differences between the flow density (whole en face) in the first session and second session with and without ET were statistically non-significant (with ET: superficial retinal OCT angiogram: first session: 54.4 ± 1.9; second session: 54.2 ± 2.1; *p* = 0.50; deep retinal OCT angiogram: first session: 59.8 ± 1.5; second session: 59.9 ± 1.5; *p* = 0.89; without ET: superficial retinal OCT angiogram: first session: 54.3 ± 2.0; second session: 54.4 ± 1,8; *p* = 0.81; deep retinal OCT angiogram: first session: 58.0 ± 3.3; second session: 58.9 ± 1.6; *p* = 0.24) (Tables 1 and 2).

**Table 1:** Mean flow density ± SD; (min- max) in the superficial OCT angiogram for each session; AD: Mean of the absolute difference between the first and second session; p Val.: *P*-value (paired t-test); CR: coefficients of repeatability (95% confidence intervals) and ICC: intraclass correlation coefficient (95% confidence intervals) with and without eye tracker

		Vessel density 1	*p* Value	Vessel density 2	*p* Value	AD	*p* Value	CR 95% CI	ICC 95% CI
		Mean ± SD (min -max)	with vs. without eye tracker	Mean ± SD (min -max)	with vs. without eye tracker	Mean ± SD	density 1 vs. density 2	(lower limit-upper limit)	(lower limit-upper limit)
whole en face	with eye tracker	54.4 ± 1.9 (51.3–56.8)	0.79	54.2 ± 2.1 (48.5–57.3)	0.55	1.3 ± 0.9	0.50	3.07 (1.88–4.26)	0.70 (0.37–0.87)
	without eye tracker	54.3 ± 2.0 (49.0–56.6)		54.4 ± 1.8 (51.5–58.0)		1.3 ± 1.1	0.81	3.33 (2.04–4.62)	0.62 (0.25–0.83)
fovea	with eye tracker	32.7 ± 4.1 (23.7–38.3)	0.61	32.7 ± 3.9 (21.9–39.6)	0.21	1.5 ± 1.2	0.98	3.88 (2.38–5.38)	0.88 (0.72–0.95)
	without eye tracker	33.0 ± 4.0 (22.8–41.1)		33.2 ± 3.7 (22.6–38.6)		1.4 ± 1.1	0.61	3.50 (2.15–4.86)	0.89 (0.75–0.96)
parafovea	with eye tracker	56.2 ± 2.2 (52.5–60.0)	0.72	56.1 ± 2.4 (49.7–59.7)	0.99	1.4 ± 0.9	0.74	3.37 (2.06–4.67)	0.72 (0.41–0.88)
	without eye tracker	56.1 ± 2.1 (52.0–59.1)		56.1 ± 2.1 (52.2–59.9)		1.5 ± 1.0	0.98	3.60 (2.20–4.99)	0.62 (0.25–0.83)
temporal	with eye tracker	54.5 ± 2.3 (50.8–59.1)	0.50	54.5 ± 2.7 (48.2–58.1)	.034	1.2 ± 1.1	0.97	3.31 (2.03–4.59)	0.77 (0.51–0.90)
	without eye tracker	54.8 ± 2.5 (50.1–58.2)		54.9 ± 2.4 (50.9–59.5)		1.8 ± 1.3	0.79	4.36 (2.67–6.05)	0.59 (0.22–0.82)
superior	with eye tracker	57.6 ± 2.4 (54.6–63.0)	0.36	57.6 ± 2.2 (53.5–61.0)	0.44	1.4 ± 1.1	0.88	3.53 (2.17–4.90)	0.69 (0.36–0.86)
	without eye tracker	57.2 ± 2.4 (52.0–61.0)		57.1 ± 2.9 (51.6–62.6)		1.5 ± 1.3	0.82	3.92 (2.40–5.44)	0.72 (0.41–0.88)
nasal	with eye tracker	55.1 ± 2.6 (50.3–59.9)	0.75	54.3 ± 2.4 (48.6–58.3)	0.17	1.5 ± 0.8	0.04	3.10 (1.90–4.30)	0.81 (0.57–0.92)
	without eye tracker	54.9 ± 2.8 (49.9–58.8)		55.0 ± 2.0 (51.2–59.1)		2.5 ± 1.7	0.89	6.01 (3.68–8.33)	0.18 (− 0.27–0.57)
inferior	with eye tracker	57.8 ± 2.7 (52.0–61.6)	0.47	57.5 ± 3.5 (47.3–62.0)	0.74	1.9 ± 1.4	0.69	4.65 (2.85–6.45)	0.71 (0.39–0.87)
	without eye tracker	57.5 ± 2.7 (53.2–61.6)		57.3 ± 2.9 (49.9–61.8)		2.4 ± 1.8	0.78	5.94 (8.25–3.64)	0.40 (− 0.04–0.71)

**Table 2:** Mean flow density ± SD; (min- max) in the deep OCT angiogram for each session; AD: Mean of the absolute difference between the first and second session; *P*-value: paired t-test; CR: coefficients of repeatability (95% confidence intervals) and ICC: intraclass correlation coefficient (95% confidence intervals) with and without eye tracker

		Vessel density 1	*p* Value	Vessel density 2	*p* Value	AD	*p* Value	CR 95% CI	ICC 95% CI
		Mean ± SD (min -max)	with vs. without eye tracker	Mean ± SD (min -max)	with vs. without eye tracker	Mean ± SD	density 1 vs. density 2	(lower limit-upper limit)	(lower limit-upper limit)
whole en face	with eye tracker	59.8 ± 1.5 (56.8–62.5)	0.02	59.9 ± 1.5 (57.7–62.8)	0.01	1.4 ± 1.0	0.89	3.51 (2.15–4.86)	0.3 (−0.15–0.65)
	without eye tracker	58.0 ± 3.3 (48.2–62.2)		58.9 ± 1.6 (54.6–61.3)		2.1 ± 3.0	0.24	6.92 (4.24–9.60)	0.07 (− 0.37–0.49)
fovea	with eye tracker	31.1 ± 5.8 (20.9–43.7)	0.74	31.4 ± 5.2 (22.1–45.2)	0.90	1.6 ± 1.1	0.49	3.79 (2.32–5.26)	0.94 (0.85–0.98)
	without eye tracker	31.3 ± 5.5 (21.7–44.2)		31.3 ± 5.9 (18.7–45.6)		2.6 ± 2.1	0.98	6.59 (4.04–9.15)	0.83 (0.61–0.93)
parafovea	with eye tracker	62.6 ± 1.6 (60.1–65.8)	0.01	62.5 ± 2.0 (58.6–65.4)	0.02	1.4 ± 1.1	0.81	3.56 (2.18–4.94)	0.50 (0.08–0.77)
	without eye tracker	60.7 ± 3.3 (53.4–65.6)		61.6 ± 2.1 (56.9–64.5)		2.2 ± 2.3	0.22	6.07 (3.72–8.42)	0.37 (− 0.08–0.69)
temporal	with eye tracker	60.7 ± 2.3 (55.5–64.5)	0.35	60.8 ± 2.2 (57.1–65.6)	0.82	2.0 ± 1.6	0.86	5.11 (7.09–3.13)	0.33 (− 0.12–0.67)
	without eye tracker	59.9 ± 3.1 (52.4–65.1)		60.9 ± 2.3 (57.1–64.8)		2.3 ± 2.4	0.18	6.20 (3.80–8.60)	0.35 (− 0.10–0.68)
superior	with eye tracker	64.0 ± 1.8 (60.6–69.0)	0.01	64.4 ± 1.8 (60.0–67.6)	< 0.01	1.7 ± 1.2	0.48	4.13 (2.53–5.73)	0.49 (0.08–0.76)
	without eye tracker	62.0 ± 3.7 (54.4–68.0)		62.5 ± 2.6 (56.9–66.1)		1.9 ± 2.2	0.43	5.74 (3.52–7.97)	0.57 (0.19–0.81)
nasal	with eye tracker	61.6 ± 1.6 (58.2–63.4)	0.01	61.0 ± 2.1 (56.9–63.9)	0.53	1.5 ± 1.1	0.14	3.43 (2.10–4.76)	0.55 (0.16–0.80)
	without eye tracker	59.2 ± 4.2 (50.7–65.0)		60.6 ± 3.0 (53.9–67.6)		3.6 ± 3.3	0.19	9.22 (5.65–12.79)	0.16 (− 0.29–0.55)
inferior	with eye tracker	64.2 ± 1.8 (60.5–67.9)	0.05	64.2 ± 2.6 (57.8–67.9)	0.01	1.8 ± 1.6	0.99	4.71 (2.88–6.53)	0.41 (− 0.03–0.72)
	without eye tracker	61.9 ± 5.3 (45.0–69.4)		62.4 ± 2.8 (53.6–66.4)		3.6 ± 4.3	0.68	11.07 (6.78–15.35)	0.11 (0.34–0.52)

The flow density, the mean of the absolute difference (AD) between the first and second session, the CR and the ICC for the two sessions with and without ET are shown in Table 1 (superficial retinal OCT angiogram) and in Table 2 (deep retinal OCT angiogram). There was no significant difference in the coefficients of repeatability (CR) for measurements with and without ET in the superficial retinal OCT angiogram (adjusted *p*-value = 0.176), whereas for the deep retinal OCT angiogram, the CR for measurements with ET was significantly lower than for measurements without ET (adjusted *p*-value = 0.008).

**Fig. 1:**
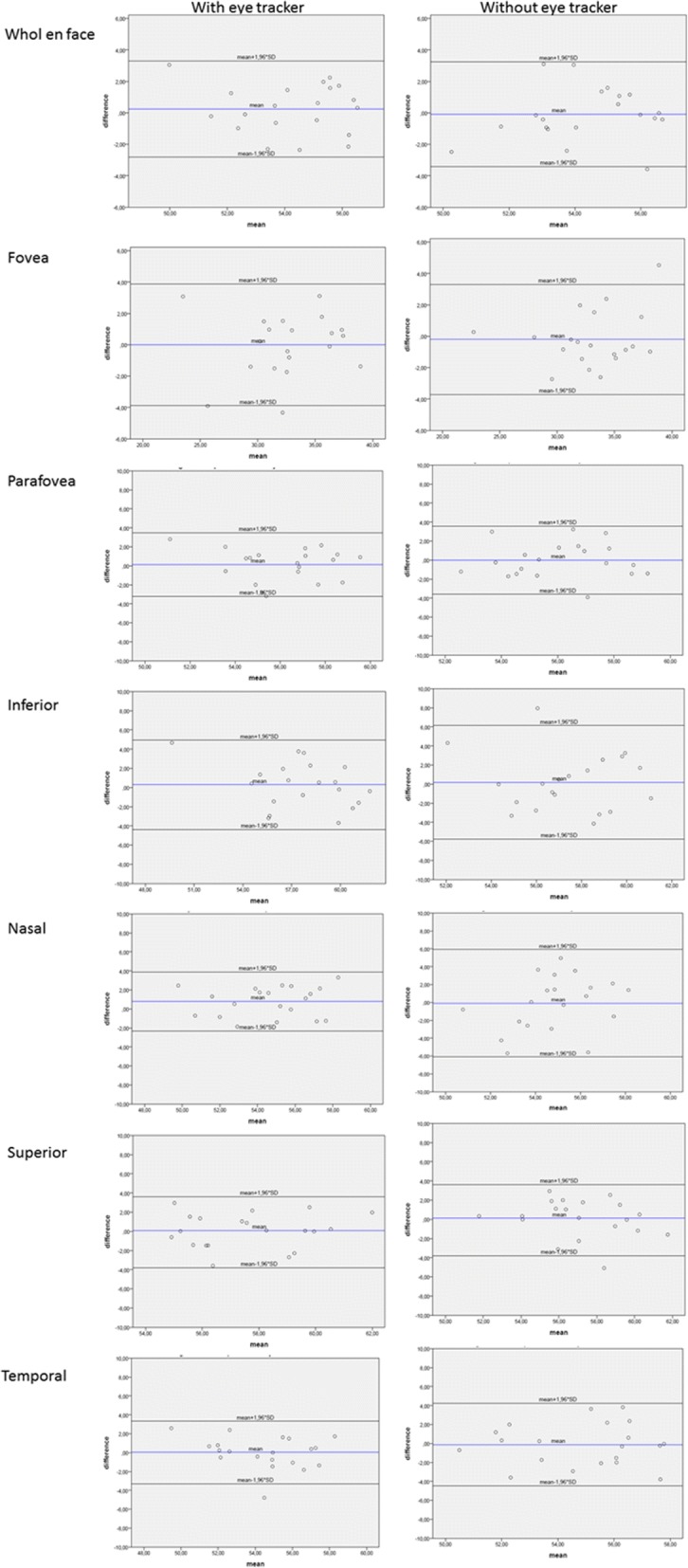
Bland-Altman plots showing the level of agreement for the superficial retinal layer with and without eye tracker. Blue line represents the mean difference; black lines represent the limits of agreement

**Fig. 2:**
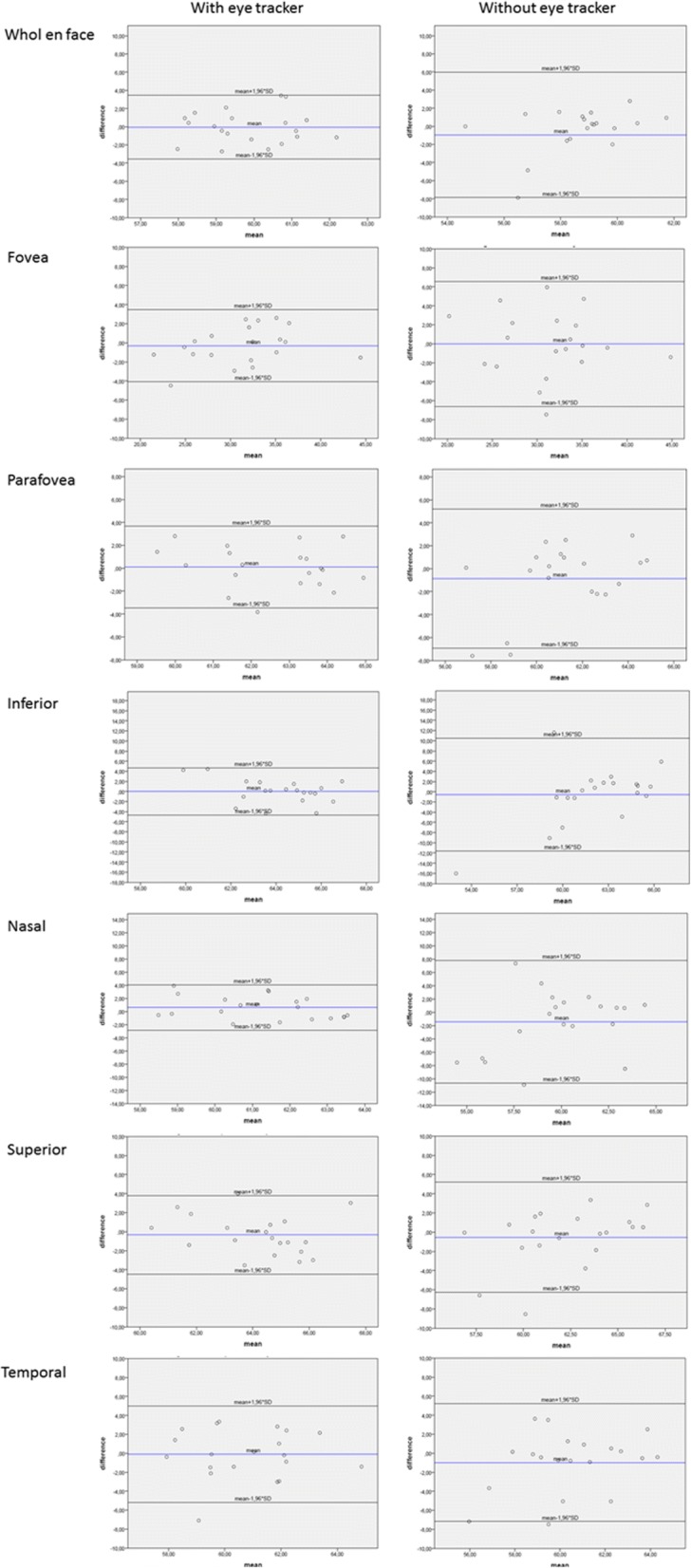
Bland-Altman plots showing the level of agreement for the deep OCT angiogram with and without eye tracker. Blue line represents the mean difference; black lines represent the limits of agreement

Bland-Altman plots for the superficial (Fig. 1) and the deep (Fig. 2) retinal OCT angiograms demonstrate the agreement between the two sessions for measurements with and without eye tracker.

## Discussion

OCT angiography is a noninvasive imaging technique that enables visualization of retinal vessels in the superficial and deep vascular plexuses without intravenously injected dye. A very interesting feature of OCT angiography is the possibility of blood flow quantification in the different retinal layers. The quantification of retinal or choroidal blood flow and the analysis of repeatability and reproducibility of blood flow measurements have attracted increasing interest over the last two years. The new approach has been described, using various OCT angiography devices, in healthy subjects and in patients with different ocular and systemic diseases [9–16, 21].

The split-spectrum amplitude-decorrelation angiography (SSADA) algorithm is used by the RTVue XR Avanti to extract the OCT angiography information. This device is used to visualize retinal or choroidal vessels and to quantify blood flow in the macula and in the optic nerve head without using an eye-tracking system, and a number of studies in the literature have evaluated its utility [9, 10, 12–17]. New software provided with the device enables activation of an ET. In the study presented here, we evaluated the repeatability of flow density measurements and the impact of the eye-tracking system on the repeatability of FD measurements in the different retinal layers. Especially in the deep OCT angiogram, integration of the ET in the RTVue XR Avanti device while performing OCT angiography imaging has improved the repeatability of quantification of blood flow measurements.

Coscas et al. evaluated the repeatability and reproducibility of FD measurements using the Optovue device. In this study, evaluation of the FD measurements of 135 eyes of 70 subjects (aged 19–66 years) demonstrated high inter- and intra-examiner repeatability and interexaminer reproducibility. The ICCs of FD measurements were not statistically significantly different between the two sessions or between the two examiners in either the deep or superficial capillary plexuses [9]. Al-Sheikh et al. presented a study with a similar design on 41 eyes of 21 healthy subjects (age: between 18 and 90 years old) for the NIDEK RS-3000 Advance device [11]. The CR and CV measurements in these studies are comparable to our findings, although the ICCs measured by Al-Sheik et al., especially in the deep OCT angiogram, were higher than those obtained by us. The ICC is the ratio of the intersubject component of the variance to the total variance. The higher the ratio, the better the repeatability; the variability of measurements is primarily the result of interindividual differences [22]. The differences in ICCs between these studies may be explained by the different numbers of subjects, the differences in subject age or by the inclusion of both eyes of the same subject in the evaluations reported by Al-Sheikh et al. and Coscas et al. Analysis of the Bland-Altman plots and CR readings in these studies are comparable to our results and demonstrate good repeatability of vessel density measurements with OCT angiography in healthy subjects [9, 11].

Our study was also designed to evaluate the impact of the eye-tracking system on the repeatability of the flow density measurements. On examining the Bland-Altman plots with and without eye-tracking, a considerable improvement in repeatability is apparent in the deep OCT angiogram and would be of importance when evaluating flow density in a specific sector. The difference in the CR between measurements with and without eye tracking was also only significant for the deep retinal OCT angiogram.

Different studies in the literature show that the repeatability of FD measurements in the superficial retinal OCT angiogram was higher compared with the deep OCT angiogram [11, 23]. This finding might be related to the higher resolution and image quality of the superficial plexus compared to the deep plexus. Fenner et al. found that different factors affected repeatability of FD in the deep retinal OCT angiogram including low visibility of fine vessels or the presence of motion artefact [23]. The ET technology offers an improved image quality in OCT-A imaging regarding presence of motion artifacts [24]. This would explain the more pronounced improvement in the repeatability of FD described in our study.

The FD in the deep retinal OCT angiogram was found to be altered in different ocular diseases such as glaucoma, adult-onset foveomacular vitelliform dystrophy or in patients with retinitis pigmentosa [25–27]. The OCTA technology is still in its infancy; improved repeatability of OCTA metrics would encourage ophthalmologists to evaluate this metrics in different diseases and to use them in daily clinical practice.

As our study was carried out on healthy subjects with high quality images, the impact of the eye-tracking system on the repeatability of flow density measurements in patients with different retinal diseases remain to be evaluated in further studies. The eye-tracking system will be even more valuable in such cases, due to the challenges related to poor patient fixation and motion artifacts [17].

OCT angiography technology is still under development and the integration of the eye-tracking system has an impact on the repeatability of the blood flow measurements. This needs to be considered when comparing data of different studies using the same device or comparing measurements obtained with different OCT angiography equipment.

## Conclusions

In conclusion, the integration of the eye-tracking system improved the repeatability of flow density measurements especially in the deep OCT angiogram. This should be taken into consideration when evaluating the long-term changes of flow density and comparing data from different studies and different devices. In evaluation of the long-term changes of flow density measurements in the deep OCT angiogram, it is advisable to activate the eye-tracking system.

## Abbreviations

CR: Coefficient of repeatability
ET: Eye-tracking system
FD: Flow density
ICC: Intraclass correlation coefficient
OCT: Optical coherence tomography
SSADA: Split-spectrum amplitude-decorrelation angiography

## References

[1] Makita S, Hong Y, Yamanari M, Yatagai T, Yasuno Y (2006). Optical coherence angiography. Opt Express.

[2] Leitgeb RA, Werkmeister RM1, Blatter C, Schmetterer L (2014). Doppler optical coherence tomography. Prog Retin Eye Res.

[3] Quaranta-El Maftouhi M, El Maftouhi A, Eandi CM (2015). Chronic central serous Chorioretinopathy imaged by optical coherence tomographic angiography. Am J Ophthalmol.

[4] Jia Y, Bailey ST, Wilson DJ (2014). Quantitative optical coherence tomography angiography of choroidal neovascularization in age-related macular degeneration. Ophthalmology.

[5] Powner MB, Sim DA, Zhu M, Nobre-Cardoso J (2016). Evaluation of nonperfused retinal vessels in ischemic retinopathy. Invest Ophthalmol Vis Sci.

[6] Ishibazawa A, Nagaoka T, Takahashi A (2015). Optical coherence tomography angiography in diabetic retinopathy: a prospective pilot study. Am J Ophthalmol.

[7] Alnawaiseh M, Schubert F, Nelis P (2016). Optical coherence tomography (OCT) angiography findings in retinal arterial macroaneurysms. BMC Ophthalmol.

[8] Alnawaiseh M, Rosentreter A, Hillmann A (2016). OCT angiography in the mouse: a novel evaluation method for vascular pathologies of the mouse retina. Exp Eye Res.

[9] Coscas F, Sellam A, Glacet-Bernard A (2016). Normative data for vascular density in superficial and deep capillary plexuses of healthy adults assessed by optical coherence tomography angiography. Invest Ophthalmol Vis Sci.

[10] Lupidi M, Coscas F, Cagini C (2016). Automated quantitative analysis of retinal microvasculature in normal eyes on optical coherence tomography angiography. Am J Ophthalmol.

[11] Al-Sheikh M, Tepelus TC, Nazikyan T, Sadda SR (2016). Repeatability of automated vessel density measurements using optical coherence tomography angiography. Br J Ophthalmol.

[12] Wang X, Kong X, Jiang C (2016). Is the peripapillary retinal perfusion related to myopia in healthy eyes? A prospective comparative study. BMJ Open.

[13] Liu L, Jia Y, Takusagawa HL (2015). Optical coherence tomography angiography of the Peripapillary retina in Glaucoma. JAMA Ophthalmol.

[14] Jia Y, Wei E, Wang X (2014). Optical coherence tomography angiography of optic disc perfusion in glaucoma. Ophthalmology.

[15] Wang X, Jiang C, Ko T (2015). Correlation between optic disc perfusion and glaucomatous severity in patients with open-angle glaucoma: an optical coherence tomography angiography study. Graefes Arch Clin Exp Ophthalmol.

[16] Li J, Yang YQ, Yang DY (2016). Reproducibility of perfusion parameters of optic disc and macula in rhesus monkeys by optical coherence tomography angiography. Chin Med J.

[17] Spaide RF, Fujimoto JG, Waheed NK (2015). Image artifacts in optical coherence tomography angiography. Retina.

[18] Shrout PE, Fleiss JL (1979). Intraclass correlations: uses in assessing rater reliability. Psychol Bull.

[19] Bland JM, Altman DG (2003). Applying the right statistics: analyses of measurement studies. Ultrasound Obstet Gynecol.

[20] Bland JM, Altman DG (1999). Measuring agreement in method comparison studies. Stat Methods Med Res.

[21] Wang X, Jia Y, Spain R, Potsaid B (2014). Optical coherence tomography angiography of optic nerve head and parafovea in multiple sclerosis. Br J Ophthalmol.

[22] Carpineto P, Mastropasqua R, Marchini G (2016). Reproducibility and repeatability of foveal avascular zone measurements in healthy subjects by optical coherence tomography angiography. Br J Ophthalmol.

[23] Fenner BJ, Tan GSW, Tan ACS (2018). Identification of imaging features that determine quality and repeatability of retinal capillary plexus density measurements in OCT angiography. Br J Ophthalmol Apr.

[24] Lauermann JL, Treder M, Heiduschka P (2017). Impact of eye-tracking technology on OCT-angiography imaging quality in age-related macular degeneration. Graefes Arch Clin Exp Ophthalmol.

[25] Alnawaiseh M, Schubert F, Heiduschka P, Eter N. Optical coherence tomography angiography in patients with retinitis PIGMENTOSA. Retina. 2017; 10.1097/IAE.0000000000001904. [Epub ahead of print] 10.1097/IAE.0000000000001904 29068913

[26] Alnawaiseh M, Lahme L, Müller V (2018). Correlation of flow density, as measured using optical coherence tomography angiography, with structural and functional parameters in glaucoma patients. Graefes Arch Clin Exp Ophthalmol.

[27] Treder M, Lauermann JL, Alnawaiseh M (2018). Quantitative changes in flow density in patients with adult-onset foveomacular vitelliform dystrophy: an OCT angiography study. Graefes Arch Clin Exp Ophthalmol.

